# Recombinant PRV Expressing GP3 and GP5 of PRRSV Provides Effective Protection Against Coinfection With PRV and PRRSV

**DOI:** 10.1155/tbed/4612568

**Published:** 2025-05-08

**Authors:** Ruhai Guo, Hui Li, Junda Li, Jahao Qv, Guofan Ren, Xiao Zhang, Saba Nasir, Jingnan Zhang, Chen Luo, Basit Zeshan, Yefei Zhou, Honglin Xie, Xinglong Wang

**Affiliations:** ^1^College of Veterinary Medicine, Northwest A&F University, Yangling, Shaanxi, China; ^2^Faculty of Sustainable Agriculture, Universiti Malaysia Sabah, Sandakan, Sabah, Malaysia; ^3^Department of Life Science, Nanjing Xiaozhuang University, Nanjing, Jiangsu, China; ^4^School of Animal Science and Technology, Foshan University, Foshan, Guangdong, China

**Keywords:** GP3-GP5, NADC30-like, porcine reproductive and respiratory syndrome virus, pseudorabies virus, vaccine

## Abstract

Porcine Reproductive and Respiratory Syndrome (PRRS) and Pseudorabies (PR) are highly contagious diseases caused by Porcine Reproductive and Respiratory Syndrome virus (PRRSV) and Pseudorabies virus (PRV). Due to the limited protective ability of currently commercialized vaccines against NADC30-like PRRSV and PRV variants, the pathological damage caused by coinfection of these two viruses has a significant impact on China's pig farming industry. In this study, six recombinant PRV stains with TK and gI/gE deletions and fused expression of GM-CSF and GP3 and GP5 proteins from NADC30-Like PRRSV were constructed by using the HDR-CRISPR/Cas9^D10A^ system. After assessing growth characteristics and genetic stability, four strains demonstrating stable proliferation and expression of the GM-CSF, GP3, GP5 fusion protein in BHK-21 cells were selected. Evaluation of their ability to induce specific humoral and cellular immune responses in mice led to the selection of two strains with superior immunogenic effects: rPRV-ΔTK-GP3-GP5-eGFP-ΔgI/gE-mCHERRY-B and rPRV-ΔTK-eGFP-ΔgI/gE-GP3-GP5-mCHERRY-B. These strains were found to enhance NADC30-like PRRSV and PRV-specific immune responses in piglets, reduce pathological damage, and accelerate symptom resolution. In general, PRV is a promising viral vector for expressing PRRSV genes, and the data from this study provides references for new candidate vaccines against PRRSV.

## 1. Intrduction

Porcine Reproductive and Respiratory Syndrome (PRRS), caused by Porcine Reproductive and Respiratory Syndrome Virus (PRRSV), is a major challenge for the global pig industry due to its widespread occurrence and genetic diversity. The virus emerged in 1990s in Europe, US, and China in 1996 [1, 2]. PRRSV is the member of *Arteriviridae* family and order *Nidoviridales* and this virus is single stranded positive RNA virus. PRRS infection mainly affects fattening and nursery pigs by causing severe respiratory symptoms while in sows it can also result in reproductive problems [3]. The NADC30-like strain of PRRSV-1, initially identified in the United States in 2008, was first reported in China in 2012 and quickly became widespread. [4, 5]. Reports have suggested that commonly administered commercial vaccines in China, are ineffective at defending pigs against the NADC30-like PRRSV strain [6–8]. Addressing this issue and developing targeted vaccines for the NADC30-like PRRSV strain are critical to managing the growing challenge of disease control.

The swine herpesvirus known as Pseudorabies (PRV), a member of the *Alphaherpesvirinae* subfamily of the *Herpesviridae* family, inflicts heavy losses on pig farming [9]. The Bartha-K61 vaccine effectively controlled PRV until 2011, when variant strains emerged that differ antigenically from classical strains [10, 11]. PRV is a double-stranded DNA virus that is roughly 150 kb in size and contains about 70 open reading frames (ORFs) that are responsible for encoding for 70–100 different viral proteins [12]. The large genomic size of PRV makes it easier to incorporate and eliminate a variety of virulence and nonessential genes, including TK, gI, and gE, in order to express exogenous genes [13]. The process of insertion or deletion of particular genes reduces the virulence of the virus while it does not significantly affect its immunogenicity, which is why PRV is considered a potent vector for vaccine development. [14, 15]. Such type of recombinant vaccines elicits the specific immune response against the targeted virus while the autoimmune characteristics of the vector virus remain intact.

The CRISPR/Cas9 system has proven to be one of the most effective examples of Type-II CRISPR/Cas system as it exhibits a huge potential in gene editing [16]. In this study, HDR-CRISPR/Cas9^D10A^ was used to construct several recombinant PRVs with deletions of TK, gI, and gE genes. Meanwhile, the insertion of NADC30-like PRRSV GP3 and GP5 proteins, as well as GM-CSF, which has confirmed the potential of an adjuvant for PRV virus vector vaccines in preliminary research [13]. Subsequently, evaluations and screenings were conducted based on the biological characteristics, genetic stability of these recombinant PRVs. As well as their safety and immune response capabilities demonstrated in mice and piglets. Our research offers new insights into the future prevention and control of NADC30-like PRRSV and PRV, as well as the development of vaccines.

## 2. Materials and Methods

### 2.1. Cell Culture, Viruses, and Plasmids

BHK-21 cell lines (ATCC No. CCL-10) and Marc-145 cell lines (ATCC No. CRL-12219), obtained from the American Type Culture Collection (ATCC, Manassas, VA, USA), were cultured in Dulbecco's modified Eagle's medium (DMEM; Gibco, Carlsbad, California, United States) supplemented with 10% heat-inactivated fetal bovine sera (FBS; Sigma, St. Louis, MO, USA), and 100 UI/mL streptomycin and penicillin (Thermo Fisher Scientific, Waltham, MA, USA).

NADC30-Like PRRSV strain PRRSV-XM-2020 (GenBank MZ160905.1) [17]. PRV wild-type strain PRV-SX-10 (GenBank PP097194.1), rPRV-ΔTK-eGFP, rPRV-ΔgE/gI-mCHERRY, rPRV-ΔTK-eGFP-ΔgE/gI-mCHERRY strain (TK and gE/gI genes were deleted from PRV-SX-10, eGFP, and mCHERRY expression cassette knock in) [13] were preserved at the Veterinary Medicine College in Northwest A&F University maintained at −80°C. PRV-Bartha-K61 Vaccine was purchased from Harbin Pharmaceutical Group Bio-vaccine Co., Ltd. (Harbin, China).

Plasmids pUC19 (pUC19, Addgene plasmid #50005, Cambridge, MA, USA) and pX335-dualU6-TagBFP were modified using the commercial plasmid pX335-U6-Chimeric_BB-CBh-hSpCas9n (D10A) (pX335, Addgene plasmid #42335, Cambridge, MA, USA), which contains an additional U6 promoter-driven single guide RNA (sgRNA) expression cassette along with a reporter gene TagBFP [13]. These plasmids were all stored at the Veterinary Medicine College in Northwest A&F University maintained at −80°C.

### 2.2. Construction of sgRNAs and Recombinant Transfer Plasmids

**Figure 1:**
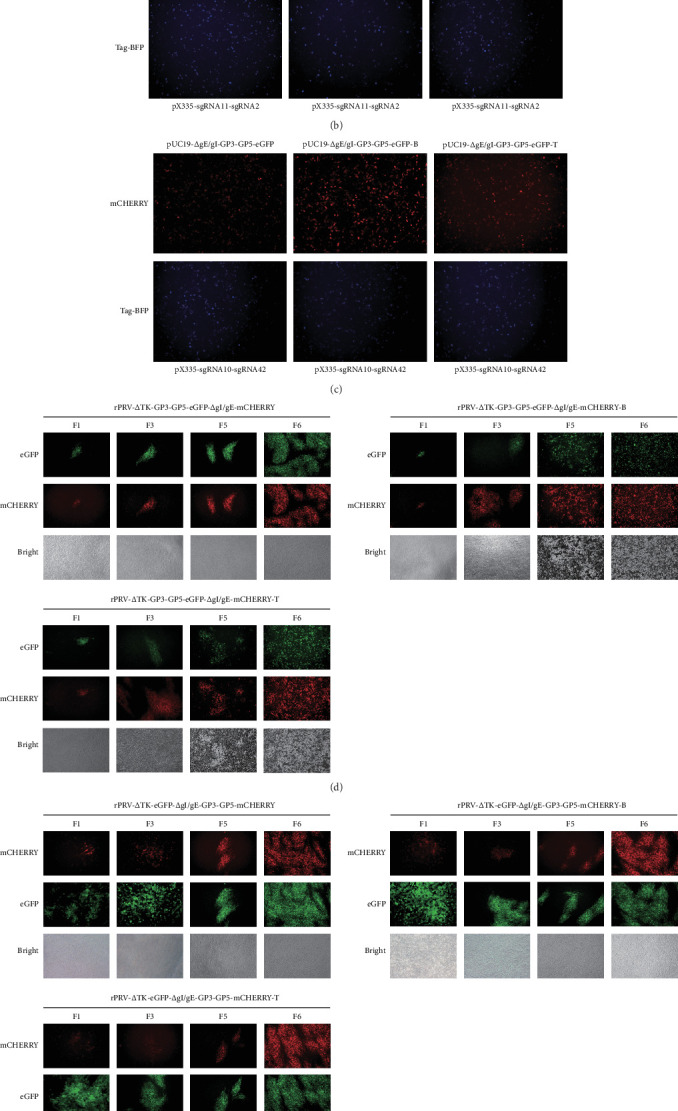
Construction and purification of recombinant PRVs. (A) Schematic diagram outlining the construction of recombinant PRVs through HDR-CRISPR/Cas9 system, (B) and (C) The fluorescence expressions of the donor plasmids and recombinant transfer plasmids were observed under the inverted fluorescence microscope at 24 h after transfection, that observing under a microscope with a magnification of 100×, (D) and (E) The purification of six recombinant PRVs, that observing under a microscope with a magnification of 100×, (F) WB Results of four stably passaged recombinant PRVs fusion protein with *β*-Tubulin as the internal reference.

An online CRISPR guide RNA designing tool (http://crispor.tefor.net/, accessed on November 16, 2022) was used to create the sgRNAs, which target the PRV genome's UL23 (TK), US7 (gI), and US8 (gE). Every sgRNA was cloned into the vector pX335-dualU6-TagBFP by inserting it into the *Bbs* I and *Bsa* I cloning sites after annealing and it was made sure that any potential off target sequence was eliminated. The Double sgRNAs pX335 plasmid was created to target the TK gene (sgRNA11, sgRNA2) and the gE/gI genes (sgRNA10, sgRNA42). The expression of TagBFP could be used to indicate the double U6 promoter-driven sgRNAs expression cassette. All sgRNAs were shown in (Supporting Table S1) and (Figure 1A).

In order to construct the recombinant transfer plasmid for TK and gE/gI deleted and GM-CSF, NADC30-like PRRSV ORF3 and ORF5 genes, eGFP, mCHERRY fusion expression cassette knock in. The homologous arms, CMV promoter and eGFP were amplified from the rPRV-ΔTK-eGFP-ΔgE/gI-mCHERRY genome. The GM-CSF coding gene (GenBank accession no. AY116504.1) was synthesized by General Biosystems (Anhui, China). The NADC30-like PRRSV ORF3 and ORF5 genes were amplified from the XM-2020 strain genome. GM-CSF, ORF3 and ORF5 are connected in three ways, seamless connection (GM-CSF-ORF3-ORF5), utilize *BmaH Ⅰ* (GM-CSF-*BmaH Ⅰ*-ORF3-*BmaH Ⅰ*-ORF5) or self-cleaving T2A peptide (GM-CSF-T2A-ORF3-T2A-ORF5), The nucleotide sequences of *BmaH Ⅰ* and T2A were added to these donor templates by PCR amplification. These three types of donor templates were obtained by fusion PCR amplification; subsequently, a T2A was added behind ORF5 by PCR amplification. The expression of fusion expression cassette could be reflected by the expression of eGFP or mCHERRY. pUC19 was released by *Hind* III and *EcoR* I and ligated with three amplified fragments above through an infusion kit (Beyotime, Shanghai, China). All the sequences of the primers are listed in (Supporting Table S1).

### 2.3. Generation of Recombinant PRVs

In a 6-well plate containing BHK-21 cells, 2 μg of the sgRNAs (sgRNA11-sgRNA2 and sgRNA10-sgRNA42) and 2 μg of the recombinant transfer plasmid were co-transfected according to the instructions included in the TurboFect transfection reagent kit (Thermo Fisher Scientific, Waltham, MA, USA). Cells were infected with rPRV-ΔTK-eGFP or rPRV-ΔgE/gI-mCHERRY at a multiplicity of infection (MOI) of 0.01 24 h after transfection. The cells were then covered with 1% low melting point agarose (HyAgarose, Piscataway, NJ, USA), until the fluorescence plaques were observed under the inverted fluorescence microscope. The recombinant PRVs were purified using the inverted fluorescence microscope and the plaque purification method in a 6-well plate. The six obtained recombinant PRVs were named rPRV-ΔTK-GP3-GP5-eGFP-ΔgI/gE-mCHERRY, rPRV-ΔTK-GP3-GP5-eGFP-ΔgI/gE-mCHERRY-B, rPRV-ΔTK-GP3-GP5-eGFP-ΔgI/gE-mCHERRY-T, rPRV-ΔTK-eGFP-ΔgI/gE-GP3-GP5-mCHERRY, rPRV-ΔTK-eGFP-ΔgI/gE-GP3-GP5-mCHERRY-B, and rPRV-ΔTK-eGFP-ΔgI/gE-GP3-GP5-mCHERRY-T.

### 2.4. Genetic Stability and Replication Kinetics of Recombinant PRVs

In order to evaluate the recombinant PRVs' genetic stability and the impact of TK or gE/gI gene knockout, we examined the presence of GM-CSF, ORF3, ORF5, TK, and gE/gI gene using polymerase chain reaction (PCR) at the F1, F10, and F30 generations. Specific primers were showed in (Supporting Table S2). The Western Blot method was then used to identify the expression of fusion proteins of recombinant PRVs. The internal reference for the primary antibodies was *β*-Tubulin monoclonal antibody (1:1000 dilution, Proteintech Group, Inc., USA), and the GP5 polyclonal antibody (1:500 dilution). After that, BHK-21 cells were infected at a MOI of 0.01 to determine the growth kinetics of the recombinant PRVs. The virus's one-step growth curve was plotted and the Reed–Muench method was used to determine the 50% tissue culture infective dose (TCID_50_).

### 2.5. Safety and Immunogenicity Assessment in Mice

A total of 80, 6-week-old, 20–23 g, female KM mice were obtained from Chengdu Dossy Experimental Animals Co. Ltd. (Chengdu, Sichuan, China), which were then assigned into eight groups. As a negative control, 100 µL of DMEM was injected intramuscularly into one group; the other seven groups were intramuscularly injected with 100 µL (1 × 10^5^ TCID_50_)) of the recombinant PRVs (including strains: rPRV-ΔTK-GP3-GP5-eGFP-ΔgI/gE-mCHERRY, rPRV-ΔTK-GP3-GP5-eGFP-ΔgI/gE-mCHERRY-B, rPRV-ΔTK-eGFP-ΔgI/gE-GP3-GP5-mCHERRY, rPRV-ΔTK-eGFP-ΔgI/gE-GP3-GP5-mCHERRY-B, and the empty vector rPRV-ΔTK-eGFP-ΔgI/gE-mCHERRY), as well as PRV-SX-10 and PRV-Bartha-K61 Vaccine. The infection method and dosage were referenced from earlier studies [13]. The mice that died were immediately necropsied, and five mice were randomly selected and euthanized in those survived groups. Brains, lungs, and livers from all groups were collected for viral load determination and hematoxylin-eosin (H&E) staining. Two weeks following the priming immunisation, the remaining mice in each group received the boosting immunisation. Sera was collected weekly throughout the experiment (7 weeks) and antibody titer was then determined.

### 2.6. Enzyme-Linked Immunosorbent Assay (ELISA)

The blood of mice in each group was collected weekly from the orbital veins using glass capillaries, ensuring their survival, post immunization. Subsequently, the serums were obtained by centrifuging the blood samples at 3000 rpm for 15 min. To evaluate the PRRSV specific GP3-GP5 antibody, a 96 well ELISA plate was coated with 30 ng GP5 protein (obtained by prokaryotic expression in our lab) at 4°C overnight and subsequent steps were consistent with earlier studies. To evaluate the PRV specific glycoprotein B (gB) antibody, commercial ELISA kits (Shanghai Hengyuan Bioengineering Co., Shanghai, China) were utilized. HRP-conjugated secondary antibody IgG changed into (HRP)-labeled goat anti-mouse IgG (diluted with PBST at 1:5000), other steps following the manufacturers' instructions.

### 2.7. Neutralizing Antibody Assay

Serum samples from the immunized mice and control groups were taken at 14 and 21 days after vaccination, and the samples were heated to 56°C in a water bath for 30 min to inactivate the antibodies. The samples were subsequently serially diluted twice using DMEM. Each sample was mixed with a 200 TCID_50_ PRV-SX-10 or NADC30-Like PRRSV-XM-2020 solution and incubated at 37°C in a 5% CO_2_ incubator for 1 h. The mixture was then added to 96-well plates with both Marc-145 or BHK-21 cells lined up, and the cells were cultured. The Reed–Muench method was used to calculate the serum antibody titers following the collection of the 72-h cytopathic effect (CPE).

### 2.8. Mouse Challenge Experiments

**Figure 2:**
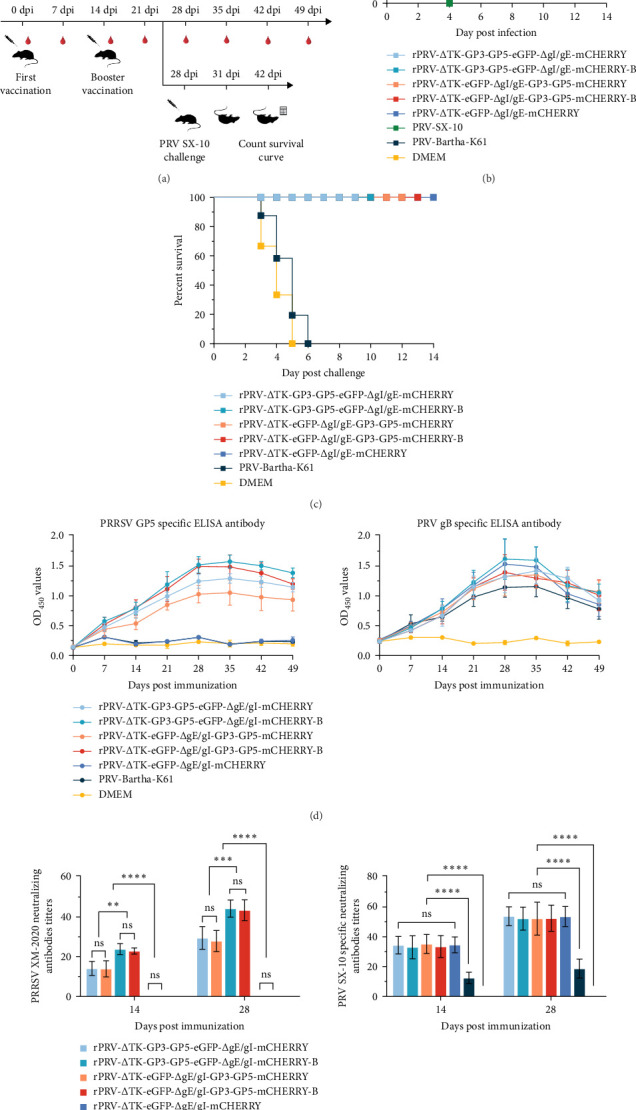
Evaluation of safety and immune protection effectiveness in mice. (A) schematic diagram outlining the immunization and challenge experiments in mice, (B) survival curve of infected mice, (C) the overall survival curves in mice challenged with PRV-SX-10, (D) the mouse PRRSV GP5 and PRV gB antibodies curves in these immunized groups, (E) the mouse neutralizing antibody titers in 14 and 28 dpi against the NADC30-Like PRRSV-XM-2020 strain and PRV-SX-10 strain (*⁣*^*∗*^*p*  < 0.05, *⁣*^*∗∗*^*p*  < 0.01, *⁣*^*∗∗∗*^*p*  < 0.001, *⁣*^*∗∗∗∗*^*p*  < 0.0001, ns indicates nonsignificant).

One hundred sixty 6-week-old, 20–23 g, female KM mice were divided into eight groups, immunized twice with recombinant PRV, PRV-Bartha-K61 vaccine, or DMEM, and infected with 100 µL (1 × 10^5^ TCID_50_) of PRV-SX-10 14 days later (refer to Figure 2A for experimental flow). The inoculation method and dosage were based on previous studies ([13]). Postinfection, mouse spleens were collected for qPCR analysis of cytokine expression all the sequences of the primers are listed in Supporting Table S2 [13], and tissue sections were stained with H&E while qPCR was used to quantify viral loads. The primers and cycle profile were determined according to (GB/T 35911–2018).

### 2.9. Immunogenicity Assessment in Piglets

**Figure 3:**
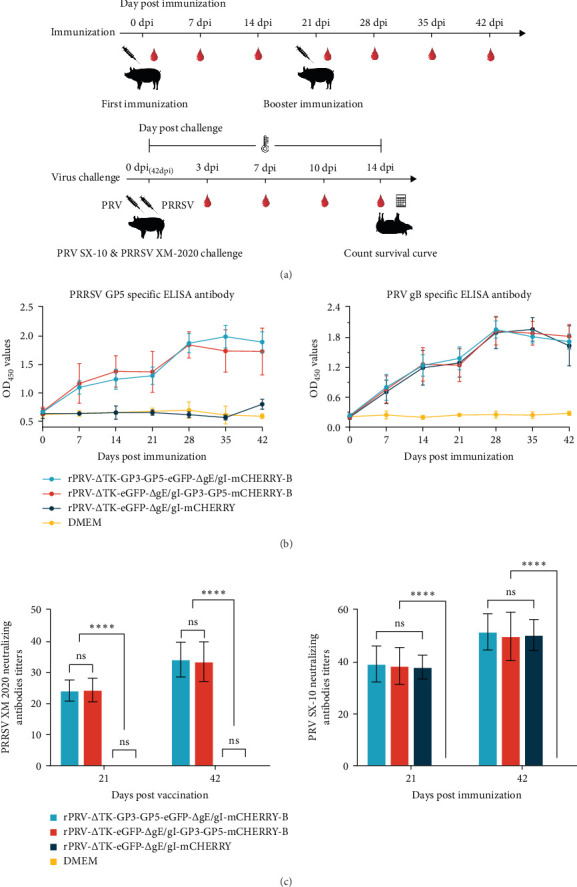
Humoral immune response of recombinant PRVs induced in piglets. (A) schematic diagram outlining the immunization and challenge experiments in piglets, (B) the PRRSV GP5 and PRV gB antibody curves in immunized piglets, (C) the piglets neutralizing antibody titers in 21 and 42 dpi against the NADC30-Like PRRSV-XM-2020 strain and PRV-SX-10 strain (*⁣*^*∗*^*p*  < 0.05, *⁣*^*∗∗*^*p*  < 0.01, *⁣*^*∗∗∗*^*p*  < 0.001, *⁣*^*∗∗∗∗*^*p*  < 0.0001, ns indicates nonsignificant).

A total of 19 weaned, castrated, 35-day-old, 9–11 kg male piglets (YORKSHIRE) were purchased from a pig farm of Hanzhong, Shaanxi, China. All the piglets were firstly kept with no treatment for a weak and then examined for PRRSV, PRV, ASFV, FMDV, PCV2, PCV3, and CSFV nucleic acid and antibodies in the lab and all of them were found to be negative. The piglets were randomly divided into five groups (Five piglets were included in each of the rPRV-ΔTK-GP3-GP5-eGFP-ΔgI/gE-mCHERRY-B group and rPRV-ΔTK-eGFP-ΔgI/gE-GP3-GP5-mCHERRY-B group, while the rPRV-ΔTK-eGFP-ΔgI/gE-mCHERRY, DMEM, and negative control groups each contained 3 piglets). The negative control group received no treatment, meanwhile the other three groups were immunized via intramuscular injection with 1 mL DMEM or 1 mL (1 × 10^5^ TCID_50_) of recombinant PRVs. The immunization method and dosage were referenced from earlier studies [18]. Following 21 days, booster shots were administered. After immunization, serum is collected and ELISA is used to detect antibodies. Neutralizing antibodies are also detected at 21 and 42 days after immunization. Peripheral blood lymphocytes are collected at 42 days after immunization for peripheral blood lymphocyte proliferation test (refer to Figure 3A for experimental flow).

### 2.10. Swine Challenge Experiments

At 21 days post boosting immunization, piglets in all groups except the negative control group were challenged via intramuscular injection with 1 × 10^6^ TCID_50_ of PRV-SX-10 and 1 × 10^7^ TCID_50_ of NADC30-Like PRRSV-XM-2020. The inoculation method and dosage for PRV were based on previous studies [18]. For PRRSV infection, the inoculation method and dosage were also referenced from previous studies [17]. However, through preliminary experiments, it was found that the virulence of the laboratory-stored PRRSV-XM2020 strain had decreased, so the infection dosage was appropriately increased. After challenge. The clinical symptoms were observed within 14 days. The dead piglets were immediately necropsied, and the survivors were euthanized 14 days later. Collect the serum of piglets before and after immunization, and use Elisa method to determine the content of cytokines in the serum. The Elisa kit is purchased from (Shanghai Enzyme-linked Biotechnology Co., Ltd. China). For piglets that died or were euthanized, necropsy should be performed immediately. The brain, lungs, liver, spleen, kidneys, tonsils, submandibular lymph nodes (SMLN), peripheral blood, as well as throat and anal swabs should be collected for viral load detection. Meanwhile, the collected tissues should be sliced and stained with H&E.

### 2.11. Histopathology Assay

The piglets that died and euthanized at 14 days after the challenge, were immediately necropsied and their organs were investigated for any kind of pathological signs and lesions. Microscopic examination was performed after collecting samples of brains, lungs, livers, spleens, and SMLNs by applying fixed staining. The guidelines for the diagnosis of clinical symptoms and histopathological changes in piglets related to PRRSV [19] and PRV [20].

### 2.12. Data Analysis

Statistical analysis was performed on the differences between the two groups using analysis of variance (ANOVA) and *t*-test. The significance was determined of relative changes through one-way ANOVA. Data were processed and visualized using GraphPad Prism 8 (GraphPad Software, Inc., USA). Significant differences between groups are denoted by asterisks: *⁣*^*∗*^*p* < 0.05, *⁣*^*∗∗*^*p* < 0.01, and *⁣*^*∗∗∗*^*p* < 0.001, *⁣*^*∗∗∗∗*^*p*  < 0.0001. Within each strain, different letters indicate significant differences at a threshold of *p* < 0.05. All data are presented as means ± standard error of the mean (SEM), derived from at least three independent experiments.

## 3. Results

### 3.1. Generation and Genetic Stability of Recombinant PRVs

Employing the HDR-CRISPR/Cas9^D10A^ system previously established in our laboratory, we have efficiently constructed six strains of recombinant PRVs that express a fusion cassette of GMCSF-GP3-GP5 (Figure 1A). Detailly, recombinant transfer plasmid and the specific sgRNAs were transfected into BHK-21. Fluorescent expression was observed at 24 h post transfection confirming the successful expression of the markers (Figure 1B, C). Subsequently, the harvested supernatants after transfection-infection were inoculated into BHK-21 cells. After several rounds of purification, the F6 generation of recombinant PRV was obtained (Figure 1D, E).

We validated the genetic stability of these six strains through 30 consecutive passages, with most of the constructs exhibiting robust genetic stability. However, two of the constructs (rPRV-ΔTK-GP3-GP5-eGFP-ΔgI/gE-mCHERRY-T and rPRV-ΔTK-eGFP-ΔgI/gE-GP3-GP5-mCHERRY-T) failed to amplify the GP3 and GP5 genes in subsequent generations, indicating a loss of these sequences. Therefore, we abandoned subsequent validation of the biological characteristics of these two constructs, including detection of exogenous proteins and measurement of growth curves. Notably, the recombinant viruses at both F10 and F30 generations did not exhibit amplification of the deleted TK and gE/gI genes, confirming the successful gene knockout. Furthermore, the replication kinetics of these recombinant viruses were comparable to that of the wild-type PRV-SX-10, indicating that the gene deletions did not impact their replication capacity. The peak virus titers of rPRV-ΔTK-GP3-GP5-eGFP-ΔgI/gE-mCHERRY-B, rPRV-ΔTK-eGFP-ΔgI/gE-GP3-GP5-mCHERRY-B, rPRV-ΔTK-GP3-GP5-eGFP-ΔgI/gE-mCHERRY, rPRV-ΔTK-eGFP-ΔgI/gE-GP3-GP5-mCHERRY, empty vector rPRV-ΔTK-GP3-GP5-eGFP-ΔgI/gE-mCHERRY and wild-type PRV-SX-10 were observed at 48 h or 60 h postinfection, reaching 10^5.667^, 10^5.746^, 10^5.889^, 10^5.657^, 10^5.867^,and 10^6.333^ TCID_50_/100 μL (Supporting Figure S1). Finally, WB results demonstrated that the four recombinant PRVs successfully expressed the fusion protein (Figure 1F).

### 3.2. Evaluation Humoral Immune Response of the Vaccine Candidate and Protection Against PRV Infection in Mice

Mice model was used to evaluated the immune characters of recombinant PRVs, the immunization and challenge protocol is presented in (Figure 2A). During the 14-day observation period postinfection, only mice infected with PRV-SX-10 succumbed to the infection between days 3 and 4. All mice in the recombinant PRV groups and the PRV-Bartha-K61 vaccine group survived throughout the experimental period without evident clinical symptoms (Figure 2B). Mice infected with PRV-SX-10 also exhibited significant tissue damage, manifesting symptoms, such as inflammatory cell infiltration in the brain, perivascular cuffing, pulmonary hemorrhage, and thickening of the alveolar walls (Supporting Figure S2). Upon subsequent challenge with PRV-SX-10, all mice in the DMEM group died within 3–5 days, while mice immunized with Bartha-K61 died between 3 and 6 days. In contrast, mice immunized with recombinant viruses survived without any notable clinical manifestations, demonstrating the efficacy of recombinant PRVs in enhancing survival rates (Figure 2C). Meanwhile, deceased mice also demonstrated evident tissue damage (Supporting Figure S3), higher mRNA levels of splenic inflammatory cytokines (IL-2, IL-4, IL-6, IL-10, and IFN-*γ*), and significantly increased viral loads in tissues (Supporting Figure S4).

The results of antibody detection indicated that mice immunized with the recombinant PRVs exhibited higher levels of antibodies against the gB protein compared to those immunized with the Bartha-K61 vaccine. Notably, in assays for NADC-30-like PRRSV-specific GP5 antibodies, the rPRV-ΔTK-GP3-GP5-eGFP-ΔgI/gE-mCHERRY-B and rPRV-ΔTK-eGFP-ΔgI/gE-GP3-GP5-mCHERRY-B groups showed improved responses compared to the rPRV-ΔTK-GP3-GP5-eGFP-ΔgI/gE-mCHERRY and rPRV-ΔTK-eGFP-ΔgI/gE-GP3-GP5-mCHERRY groups. Neutralizing antibody tests further confirmed that the immune response in those two recombinant PRV groups was more effective against both the PRV variant strains and PRRSV (Figure 2D, E).

### 3.3. Humoral Immune Response of Recombinant PRVs Induced in Piglets

Swine model was used to evaluated the immune characters of recombinant PRVs, the immunization and challenge protocol is presented in (Figure 3A). Results in (Figure 3B) showed that low levels of humoral immune responses were detected after the primary immunization, but significantly increased anti-gB and GP5 antibodies were observed after the booster immunization. GP5-specific antibodies were not detected in the DMEM and rPRV-ΔTK-eGFP-ΔgI/gE-mCHERRY groups. Similarly, anti-gB antibodies were also not detected in the DMEM group. The neutralizing antibody results were consistent with the ELISA findings (Figure 3C).

**Figure 4:**
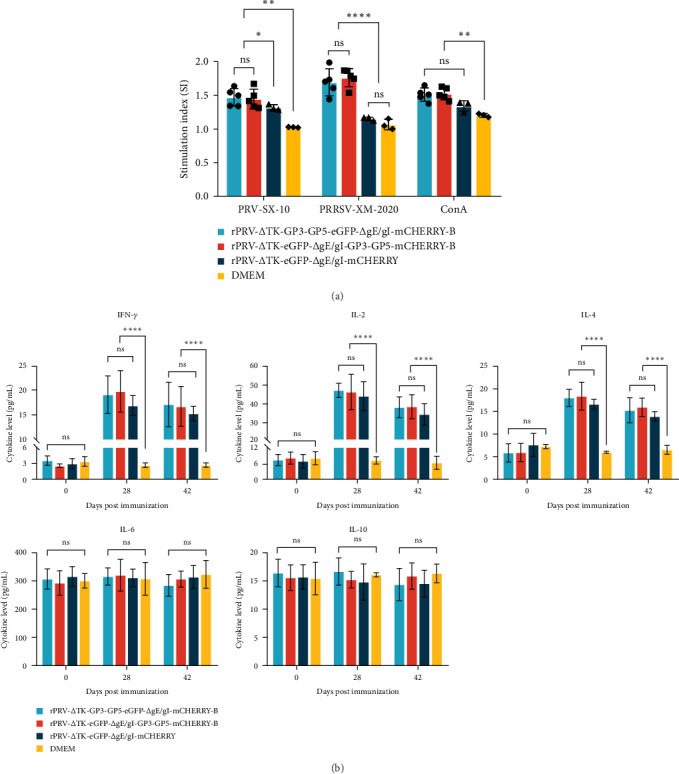
Cellular immune response of recombinant PRVs induced in piglets. (A) peripheral blood T lymphocytes specific proliferative responses assay in 21 days post booster immunization, (B) the secretion levels of serum cytokines (IL-2, IL-4, IL-6, IL-10 and IFN-*γ*), serum samples collected at 21 and 42 dpi (*⁣*^*∗*^*p*  < 0.05, *⁣*^*∗∗*^*p*  < 0.01, *⁣*^*∗∗∗*^*p*  < 0.001, *⁣*^*∗∗∗∗*^*p*  < 0.0001, ns indicates nonsignificant).

Subsequently, the results of the piglet-specific lymphocyte proliferation assay and the determination of serum cytokine secretion levels showed that the proliferation index (SI value) (Figure 4A) and the secretion levels of IL-2, IL-4, and IFN-*γ* (Figure 4B) in the rPRV-ΔTK-GP3-GP5-eGFP-ΔgI/gE-mCHERRY-B and rPRV-ΔTK-eGFP-ΔgI/gE-GP3-GP5-mCHERRY-B groups were significantly higher than those in the DMEM and rPRV-ΔTK-eGFP-ΔgI/gE-mCHERRY groups.

### 3.4. Clinical Symptoms and Cytokines Expression in Serum After Challenged With PRRSV-XM-2020 and PRV-SX-10

**Figure 5:**
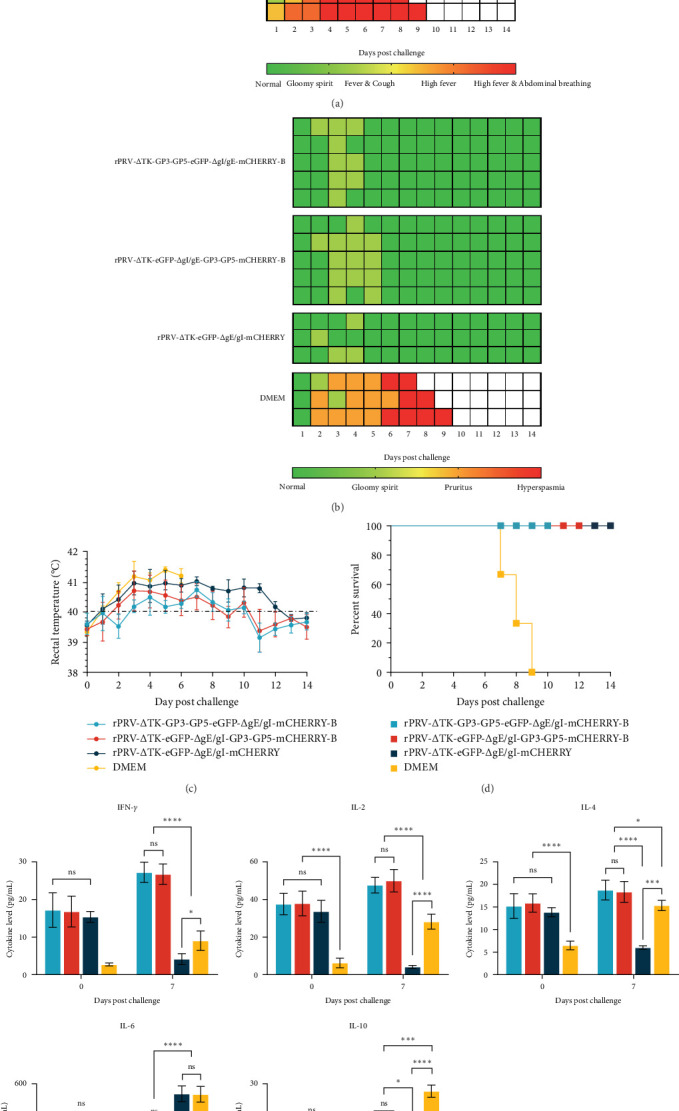
PRV and PRRSV challenge experiment for recombinant PRVs immunized piglets. (A) the changes in clinical symptoms of PRRSV (Primarily exhibiting respiratory symptoms.) were observed 14 dpc, (B) the changes in clinical symptoms of PRV (Primarily exhibiting neurological symptoms.) were observed 14 dpc, (C) the survival curves in piglets challenged with PRV-SX-10 and PRRSV-XM-2020, (D) the changes in body temperature were observed 14 dpc, (E) the secretion levels of serum cytokines, serum samples collected at 0 and 7 days post challenged (*⁣*^*∗*^*p*  < 0.05, *⁣*^*∗∗*^*p*  < 0.01, *⁣*^*∗∗∗*^*p*  < 0.001, *⁣*^*∗∗∗∗*^*p*  < 0.0001, ns indicates nonsignificant).

The clinical symptoms of piglets in various groups within 14 days after coinfection with PRRSV and PRV are depicted in (Figure 5A, B). Piglets in the rPRV-ΔTK-GP3-GP5-eGFP-ΔgI/gE-mCHERRY-B and rPRV-ΔTK-eGFP-ΔgI/gE-GP3-GP5-mCHERRY-B groups exhibited significantly milder respiratory symptoms and lower body temperatures (Figure 5C) postchallenge, compared to the rPRV/ΔTK-eGFP/ΔgI/gMCHERRY-B and DMEM groups. They also had a faster recovery, and did not experience the severe neurological symptoms or mortality observed in the DMEM group (Figure 5D). To further assess the disease status after challenge, we measured serum cytokine secretion levels. The results indicated that, compared to the DMEM and rPRV-ΔTK-eGFP-ΔgI/gE-mCHERRY groups, the recombinant PRV carrying GP3-GP5 demonstrated higher levels of IL-2, IL-4, and IFN-*γ* secretion upon rechallenge. However, the secretion levels of IL-6 and IL-10 were relatively lower (Figure 5E).

### 3.5. The Detection of Virus Shedding, Viremia, and Virus Loads in Tissues

**Figure 6:**
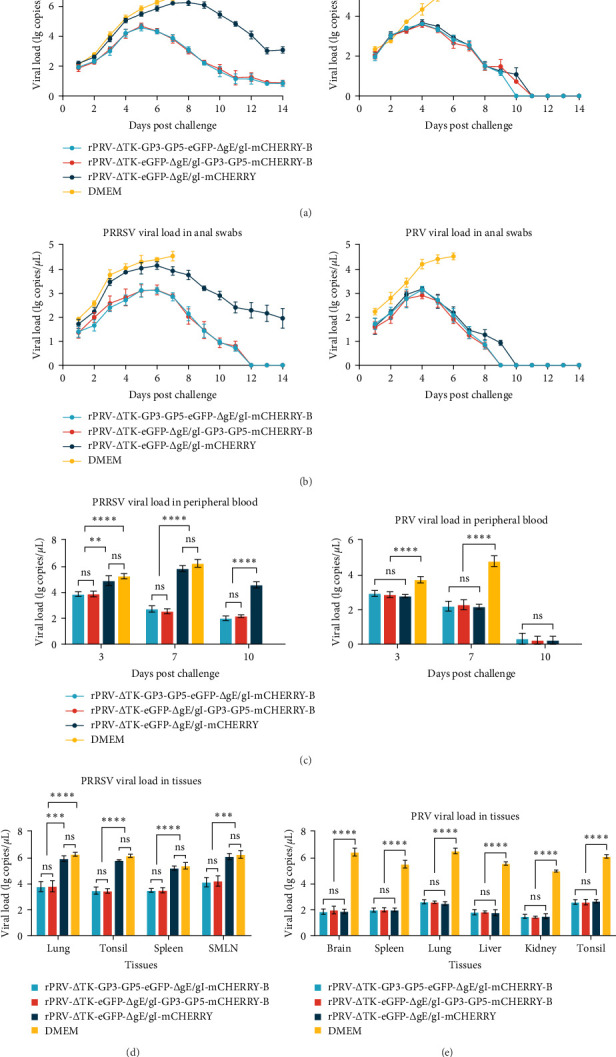
The determination of viral load post challenged by RT-qPCR or qPCR. (A) and (B) the curve of PRRSV and PRV viral load changes in throat and anal swabs during 14 dpc, (C) the PRRSV and PRV viral load detected in peripheral blood, (D) and (E) the PRRSV and PRV viral load in tissues (*⁣*^*∗*^*p*  < 0.05, *⁣*^*∗∗*^*p*  < 0.01, *⁣*^*∗∗∗*^*p*  < 0.001, *⁣*^*∗∗∗∗*^*p*  < 0.0001, ns indicates nonsignificant).

To further validate the protective ability of these two recombinant PRVs against PRV and PRRSV infections, we determined the viral loads of PRRSV and PRV in nasopharyngeal and rectal swabs, as well as in peripheral blood, postchallenge. The results showed that the PRV viral load peaked between 2 and 4 days after challenge and then decreased to below the detection limit in the rPRV-ΔTK-eGFP-ΔgI/gE-mCHERRY group, whereas the PRRSV viral load reached its highest between 2 and 8 days after challenge and continued to remain at high levels despite a subsequent decrease. In contrast, in the rPRV-ΔTK-GP3-GP5-eGFP-ΔgI/gE-mCHERRY-B and rPRV-ΔTK-eGFP-ΔgI/gE-GP3-GP5-mCHERRY-B groups, the PRRSV viral loads started to decline significantly from the 5 dpc (Figure 6A, B). It was observed that the viral load fluctuations in peripheral blood follow a similar pattern to that observed in swabs. Furthermore, at 3 and 7 days after challenge, that all recombinant PRV groups had a lower PRV viral load than the DMEM group (Figure 6C).

At the end of the 14-day observation period, the tissues of the lung, tonsil, spleen, and SMLN of groups rPRV-ΔTK-GP3-GP5-eGFP-ΔgI/gE-mCHERRY-B and rPRV-ΔTK-eGFP-ΔgI/gE-GP3-GP5-mCHERRY-B had significantly lower PRRSV viral loads than the DMEM and rPRV-ΔTK-eGFP-ΔgI/gE-mCHERRY groups (Figure 6D). Similarly, qPCR analyses of tissues including the brain, spleen, lungs, liver, kidneys, and tonsils for PRV samples revealed that all recombinant PRVs treatment groups had lower viral loads as compared to the DMEM group (Figure 6E). Moreover, the recombinant PRV groups did not differ significantly from one another, suggesting that all the strains were all equally effective.

### 3.6. The Gross and Microlesions Caused by the Coinfection of PRV and PRRSV

The postmortem examination of the piglets' tissues also confirmed the protective effects of the recombinant PRVs on the piglets. The histopathological examination revealed that the piglets in the DMEM group were found to have meningeal congestion and vascular thickening in the brain, extensive bleeding and consolidation in the lungs, and bleeding with swelling in the liver, spleen, and SMLNs. In contrast, piglets in the rPRV-ΔTK-eGFP-ΔgI/gE-mCHERRY group showed no visible damage in the brain and liver, with only slight swelling observed in the spleen. But there was still noticeable bleeding and consolidation in the lungs as well as swelling and bleeding in the SMLNs.

**Figure 7:**
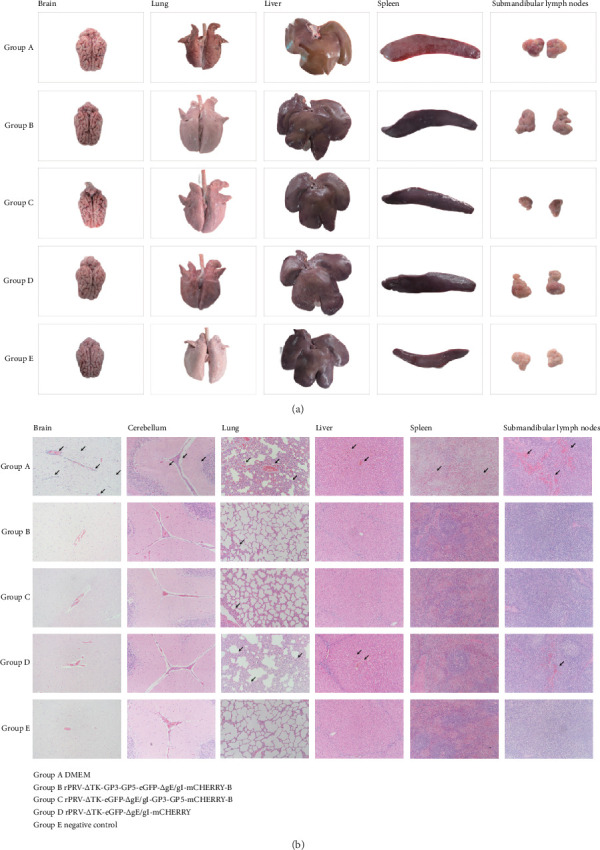
Tissue damage in piglets post challenged. (A) autopsy and examination for organ lesions immediately after the death or euthanasia in 14 days post-challenged, (B) Pathological histological analysis of tissues, that observing under a microscope with a magnification of 100×. The tissues were collected and fixed in 4% paraformaldehyde immediately after the death or euthanasia of the piglets, subsequently, HE staining was performed. The arrow points to the lesion location.

Remarkably, piglets in the rPRV-ΔTK-GP3-GP5-eGFP-ΔgI/gE-mCHERRY-B and rPRV-ΔTK-eGFP-ΔgI/gE-GP3-GP5-mCHERRY-B groups exhibited minimal tissue damage. The lungs showed only mild punctate bleeding, suggesting a significantly less severe effect than in DMEM group and rPRV-ΔTK-eGFP-ΔgI/gE-mCHERRY group. The tissues of the piglets from the negative control group appeared normal, that highlights the protective effects of the recombinant PRVs immunization in mitigating tissue damage post-challenge (Figure 7A). The original pictures of piglet organs are located in (Supporting Figure S5). At the same time, histopathological sections also confirmed the abovementioned tissue lesions. (Figure 7B).

## 4. Discussion

The NADC30-like PRRSV strain was first discovered and reported in China in 2013, isolated from the lungs of piglets immunized with the VR2332 attenuated live vaccine [6]. After more than a decade of prevalence, it has now become one of the major prevalent strains in China, and its detection rate in many provinces in China has approached or exceeded that of HP-PRRSV [21, 22]. The NADC30-like PRRSV strain is primarily characterized by its high frequency of genetic recombination, weak ability to induce immune responses, and suppression of cytokine expression, Furthermore, it exhibits lower virulence compared to HP-PRRSV [23, 24]. Previous studies have indicated that the primary approach to preventing and controlling the spread of PRRSV is through immunization with commercial vaccines, such as modified-live virus (MLV) vaccines. However, this method can only offer limited protection against the NADC30-like PRRSV strain. [6–8]. Currently, all PRRS vaccines developed in China against the NADC30-like PRRSV strain are still in the laboratory research and development stage, and no commercial NADC30-type PRRSV vaccines are available on the market. Similarly, PR is also one of the infectious diseases that have caused severe impacts on the global pig industry, especially after the emergence and prevalence of PRV variant strains in 2011. The commercialized PRV vaccine Bartha-K61 has very limited prevention and control capabilities against PRV variant strains. Moreover, coinfection with PRV and PRRSV viruses is frequently observed in breeding piglets during the fattening stage in small and medium-sized pig farms. This coinfection affects the health of the pig population, reduces the feed conversion ratio, and results in significant economic losses for pig farming enterprises. [25, 26]. This underscores the pressing need for the development of novel vaccines that specifically target NADC30-like PRRSV and PRV variant strains.

With the development of gene editing technology, PRV has gradually become a mature carrier vaccine platform, such as Cap of porcine circovirus 2(PCV2) and porcine circovirus 3(PCV3) [27], GP5 and M of PRRSV [28], VP2 and VP3 of Senecavirus A (SVA) [15], B646L and B602L of African swine fever virus (ASFV) [14], these recombinant vaccines can stimulate the body to produce a specific immune response to the target virus, without affecting the autoimmune effect of PRV. Meanwhile, according to early research, GP3 protein is the secondary outer membrane glycoprotein, which is the main structural protein and immunogenic protein of PRRSV. It can induce the production of cellular and humoral immunity [29, 30]. GP5 protein is the main outer membrane protein and is the most variable structural protein. The extracellular domain of GP5 protein is an important target for neutralizing antibodies, which makes GP5 protein highly immunogenic and has a strong ability to induce the production of neutralizing antibodies [31, 32]. The fusion expression of GP3-GP5 can induce more significant cellular immune response and humoral immune response as compared to the separate expression of GP3 or GP5 [33]. Furthermore, the fusion expression of GP3-GP5 has also been reported as the potential immunization strategy against life threatening viral infections [33, 34]. To enhance the immunogenicity of the vaccine, we introduced Granulocyte-Macrophage Colony-Stimulating Factor (GM-CSF) as a cytokine adjuvant, according to early research. GM-CSF is a cytokine produced by immune cells, such as monocytes, macrophages, and activated T and B cells, activates macrophages into M1-like proinflammatory cells and promotes the production of chemotactic and proinflammatory factors [35, 36]. Meanwhile, studies have also shown that the introduction of GM-CSF can enhance the immune effect of PRV vectors and GP3-GP5 fusion proteins [13, 34].

In this study, six strains of recombinant PRVs were engineered to fusion express GM-CSF, GP5, and GP3 proteins, with deletions of TK, gI, and gE genes. Early studies have shown that the deletion of these two loci does not affect the immunogenic properties of PRV itself, and the expression cassettes of foreign proteins inserted at these two loci have also been confirmed to stably exist and express [13]. These proteins were linked in three different configurations and inserted at two distinct genomic sites. In addition, recombinant PRVs capable of carrying exogenous genes were passaged up to 30 generations in BHK-21 cells, during which four recombinant PRV stains exhibited good stability and maintained growth kinetics similar to those of rPRV-ΔTK-eGFP-ΔgE/gI-mCHERRY. However, partial deletions of the inserted exogenous genes occurred in the rPRV-ΔTK-GP3-GP5-eGFP-ΔgI/gE-mCHERRY-T and rPRV-ΔTK-eGFP-ΔgI/gE-GP3-GP5-mCHERRY-T during passage, presumably due to deletions in the viral genome caused by the flanking repeat sequences T2A [37]. This highlights the challenges in maintaining genetic stability in viral vectors and underscores the need for careful design and evaluation of insertion sites to optimize vaccine efficacy and stability.

The safety and immunogenicity of PRV recombinant vaccine was firstly evaluated using a mouse model as despite not being the original host of PRV, mice exhibit higher sensitivity than natural host of virus, the piglets. It was observed that the recombinant PRVs demonstrated reliable safety as compared to that of Bartha-K61 vaccine. The recombinant PRVs demonstrated a significantly higher capacity to elicit neutralizing antibodies against PRV-SX-10 in comparison to Bartha-K61. Furthermore, the recombinant PRVs exhibit the capability to elicit an elevated level of GP5 antibodies and neutralizing antibodies specifically targeting NADC30-like PRRSV. Meanwhile, mice immunized with rPRV-ΔTK-eGFP-ΔgE/gI-mCHERRY and the Bartha-K61 vaccine showed significantly lower secretion of IL-2, IL-4, and IFN-*γ*, when compared to those immunized with recombinant PRVs carrying GM-CSF. This supports the results of earlier studies that suggest Th1-type T cell responses and B cell immunity are improved by the acquisition and activation of myeloid cells by GM-CSF, which increases proinflammatory responses [38]. Such enhancement bolsters the organism's capacity to eradicate pathogens, highlighting the pivotal role of GM-CSF in modulating immune dynamics [39].

The ability of rPRV-ΔTK-GP3-GP5-eGFP-ΔgI/gE-mCHERRY-B and rPRV-ΔTK-eGFP-ΔgI/gE-GP3-GP5-mCHERRY-B to induce GP5 antibodies and neutralize NADC30-like PRRSV in mice was found to be stronger than the other strains. Consequently, these strains were selected for further experimental use in immunizing piglets, to continue evaluating their potential in providing robust protection against viral infections. To better align with practical production scenarios, this study referenced the commonly used PRV immunization program in pig farms. To avoid interference from maternal antibodies, the first injection of the PRV live vaccine is typically administered to piglets at 35–70 days of age in pig farms. In this study, piglets at 35 days of age were selected for immunization. During the immunogenicity assessment trials with piglets, the recombinant PRVs displayed analogous capabilities in eliciting humoral and Cellular immune response, mirroring the findings obtained from mouse trials. Since both PRRSV-XM-2020 [17] and PRV-SX-10 (Result not published) exhibit low pathogenicity that is not fatal to older piglets. Meanwhile, coinfection with PRV and PRRSV frequently occurs in pig farms, causing more severe losses compared to infections with PRV or PRRSV alone [26, 40]. In order to simulate the coinfection scenario of PRRSV and PRV, our study utilized a challenge with both PRRSV-XM-2020 and PRV-SX-10, which was administered 21 days after the booster immunization. To simulate the coinfection scenario of PRRSV and PRV, our study employed a challenge with both PRRSV-XM-2020 and PRV-SX-10, administered 21 days after the boosting immunization. Within the 14-day observation period after the virus challenge, piglets in the DMEM group exhibited severe respiratory and neurological symptoms and all died on the 9 dpc. Similarly, the rPRV-ΔTK-eGFP-ΔgE/gI-mCHERRY group also displayed severe respiratory symptoms, though onset occurred later than in the DMEM group. By contrast, the recombinant PRVs groups showed milder symptoms and a faster recovery. The results of virus excretion, viremia, tissue virus load, autopsies and histological observations also reflect that the immunization with recombinant PRVs can mitigate the pathological damage caused by PRV and NADC30-Like PRRSV infections in piglets, and accelerate the resolution of symptoms.

The changes in cytokine levels postchallenge may provide some insights. The observed trend of change may be related to the immunosuppressive effect of NADC30-like PRRSV, which inhibits the expression of cytokines and weakens the body's defense against pathogens. [23, 24]. Notably, the immunosuppression could lead to enhanced pathogenicity of PRV and potentially allowing other pathogens encountered in farming practices to infect piglets more easily, thus causing severe tissue damage or even death [25, 26]. Notably, piglets in the rPRV-ΔTK-GP3-GP5-eGFP-ΔgI/gE-mCHERRY-B and rPRV-ΔTK-eGFP-ΔgI/gE-GP3-GP5-mCHERRY-B groups exhibited relatively stable cytokine secretion levels after challenge. The stable cytokine response underlines the effective modulation of immune dynamics, providing a robust defense mechanism against viral infections. Certainly, this study also has limitations, such as a small experimental sample size and the presence of fluorescent protein in the virus strains. In the future, our research group will continue to conduct research to improve the study outcomes. We hope that the data from this study can provide some insights for the development of vaccines against PRV and PRRSV.

## 5. Conclusion

In this study, six recombinant PRVs with TK and gI/gE deletions and fused expression of GM-CSF and NADC30-Like PRRSV GP3 and GP5 proteins were constructed using the HDR-CRISPR/Cas9^D10A^ system. By evaluating its growth characteristics, genetic stability, and evaluating its safety and immunogenicity against PRV and NADC30 Like PRRSV in mice. Two recombinant PRVs with better immune effects were selected: rPRV-ΔTK-GP3-GP5-eGFP-ΔgI/gE-mCHERRY-B and rPRV-ΔTK-eGFP-ΔgI/gE-GP3-GP5-mCHERRY-B. They can simultaneously induce piglets to produce higher levels of NADC30-Like PRRSV and PRV specific humoral and cellular immune responses, reduce the pathological damage caused by PRV and NADC30-Like PRRSV infection to piglets, and accelerate the resolution of symptoms. These results provide new insights for the control of PRRS and PR in China as well as the development of novel vaccines.

## Data Availability

The data that support the findings of this study are available from the corresponding author upon reasonable request.

## Supporting Information

Supporting Information 1 Figure S1: Identification and growth properties of recombinant PRVs. (A) PCR identification results of recombinant PRVs. (B) The one-step growth curves of the PRVs.

Supporting Information 2 Figure S2: The tissue damage in mice following immunization with recombinant PRVs.

Supporting Information 3 Figure S3: The tissue damage in mice after challenged with PRV-SX-10.

Supporting Information 4 Figure S4: The alterations in mRNA levels of cytokines and the alterations in viral load in mice (A) The expression levels of cytokines. (B) The viral load in the livers, lungs, and brains (Statistical significance was determined using two-way ANOVA in GraphPad Prism. *⁣*^*∗*^*p* < 0.05, *⁣*^*∗∗*^*p* < 0.01, *⁣*^*∗∗∗*^*p* < 0.001, *⁣*^*∗∗∗∗*^*p* < 0.0001, ns indicates nonsignificant).

Supporting Information 5 Figure S5: Original image of organs from piglet autopsy

Supporting Information 6 Table S1: Sequences of sgRNAs and primers for recombinant transfer plasmids construction.

Supporting Information 7 Table S2: The primes of PCR for detection genetic stability of recombinant PRVs and RT-qPCR for detection of cytokines.
