# Supplementary figures and images for: Recombinant PRV Expressing GP3 and GP5 of PRRSV Provides Effective Protection Against Coinfection With PRV and PRRSV
Source: Transbound Emerg Dis. 2025 May 8;2025:4612568. doi: 10.1155/tbed/4612568 (PMC12081148; doi:10.1155/tbed/4612568)

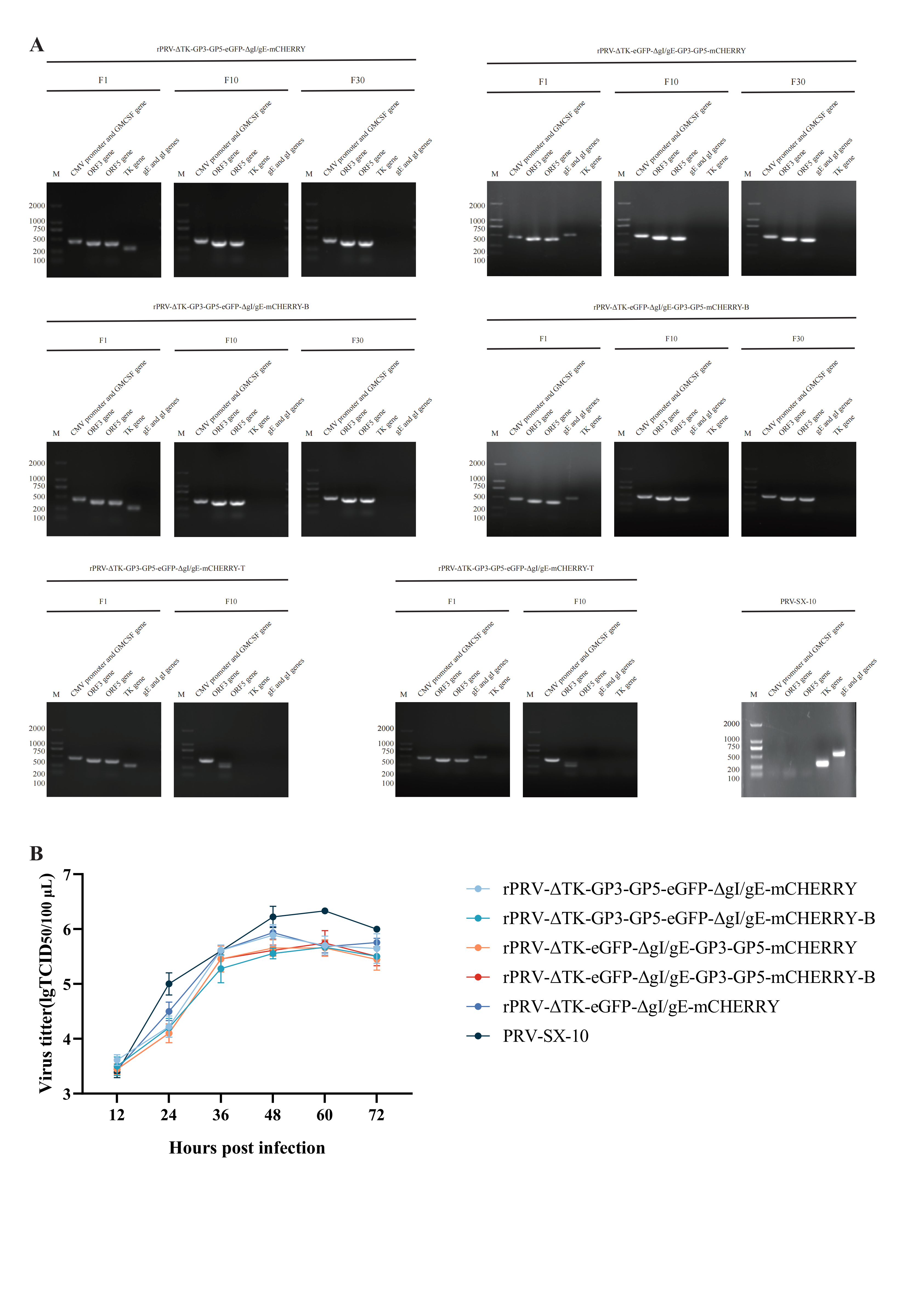

Supplement: Supporting Information 1 — Figure S1: Identification and growth properties of recombinant PRVs. (A) PCR identification results of recombinant PRVs. (B) The one-step growth curves of the PRVs. [file 4612568.f1.tif]

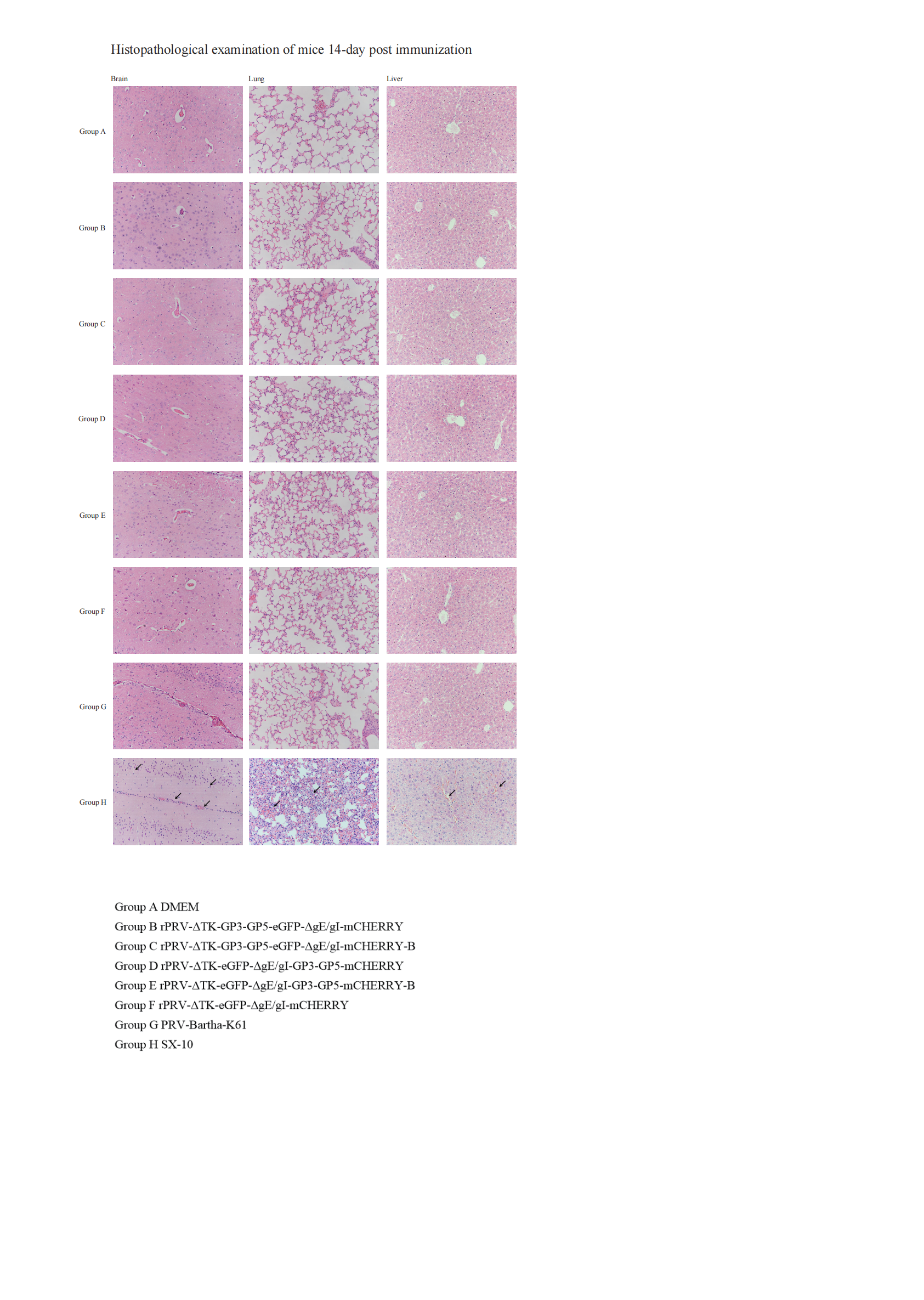

Supplement: Supporting Information 2 — Figure S2: The tissue damage in mice following immunization with recombinant PRVs. [file 4612568.f2.tif]

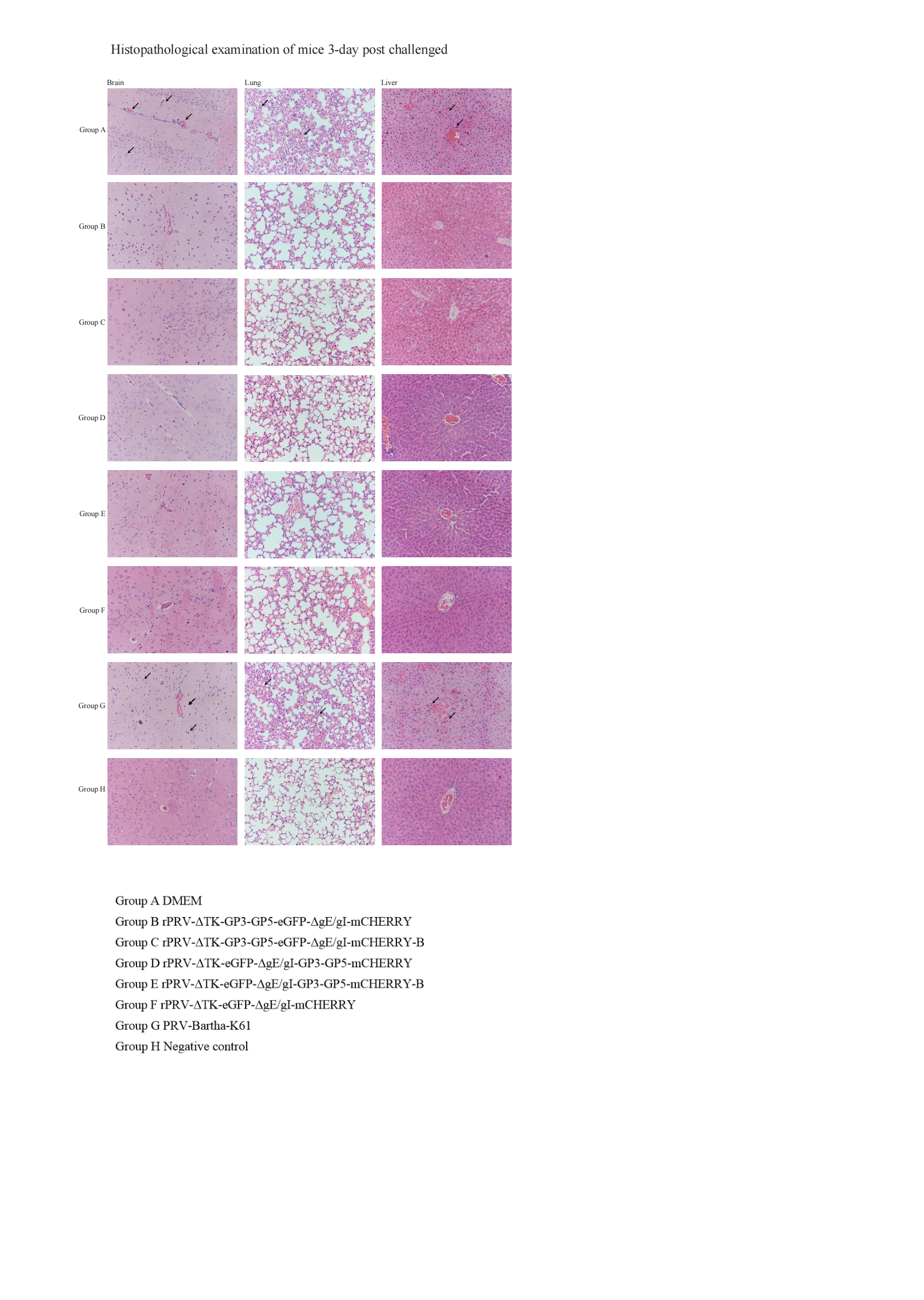

Supplement: Supporting Information 3 — Figure S3: The tissue damage in mice after challenged with PRV-SX-10. [file 4612568.f3.tif]

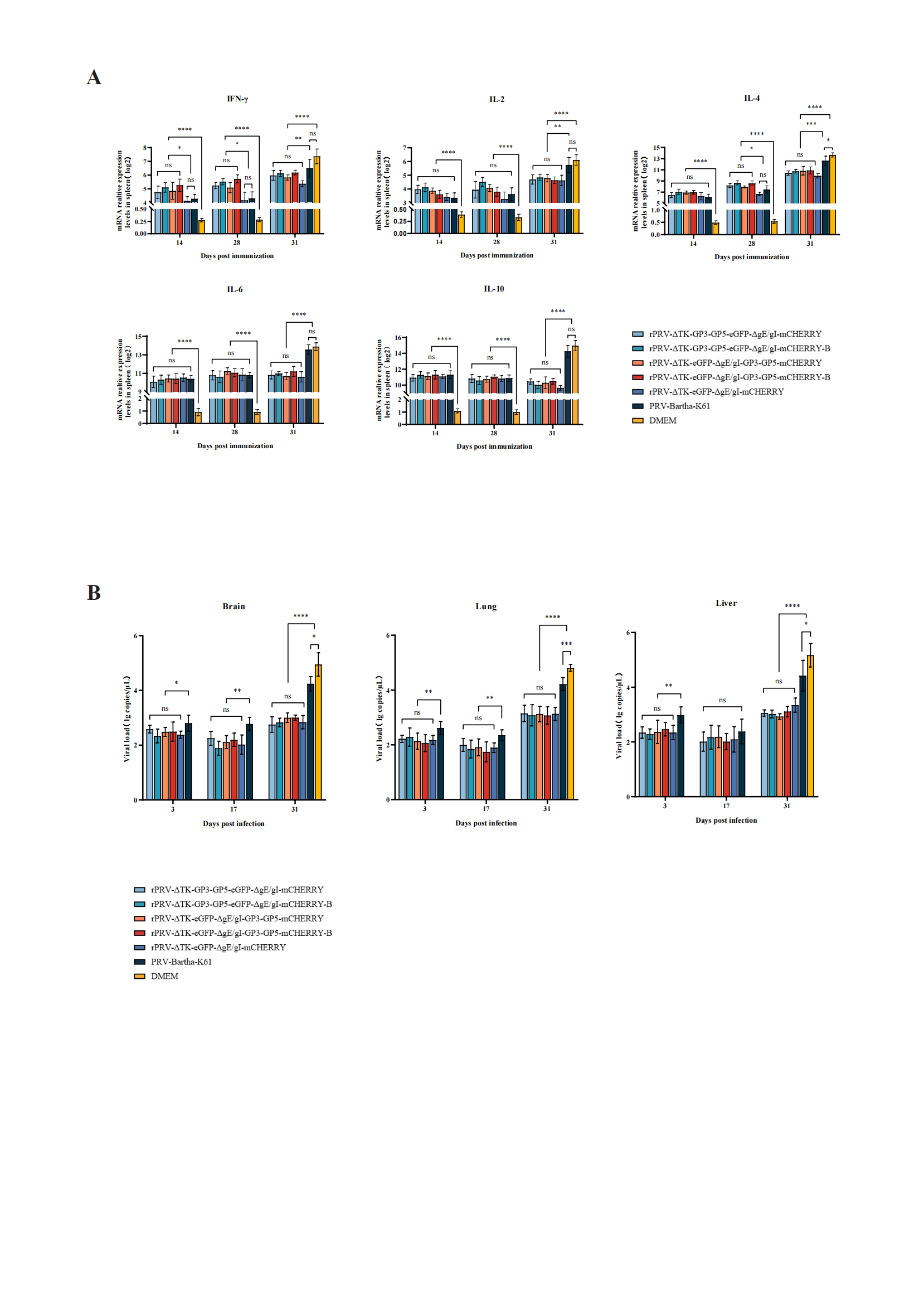

Supplement: Supporting Information 4 — Figure S4: The alterations in mRNA levels of cytokines and the alterations in viral load in mice (A) The expression levels of cytokines. (B) The viral load in the livers, lungs, and brains (Statistical significance was determined using two-way ANOVA in GraphPad Prism. ⁣∗p < 0.05, ⁣∗∗p < 0.01, ⁣∗∗∗p < 0.001, ⁣∗∗∗∗p < 0.0001, ns indicates nonsignificant). [file 4612568.f4.tif]

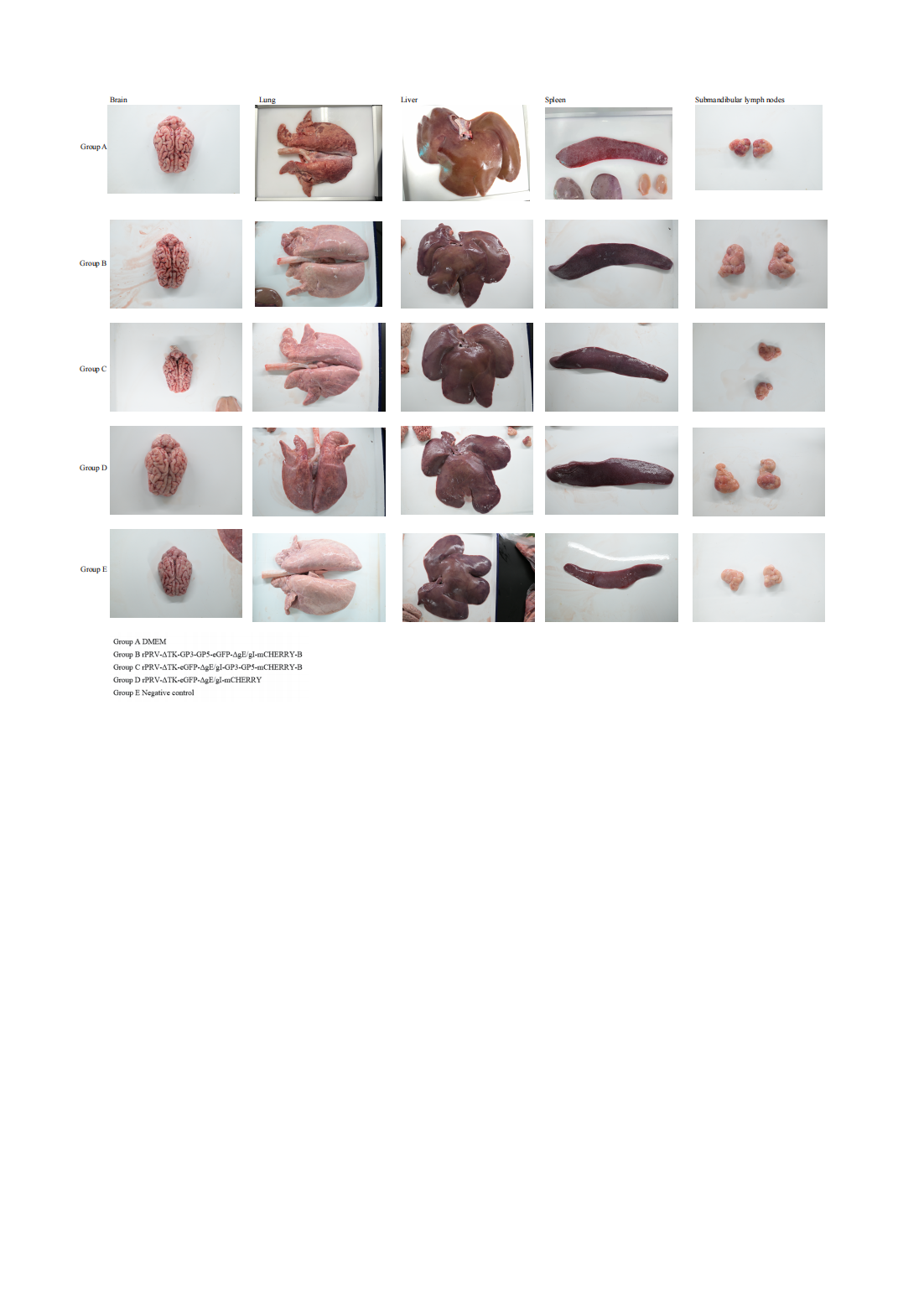

Supplement: Supporting Information 5 — Figure S5: Original image of organs from piglet autopsy [file 4612568.f5.tif]
