# Supplementary material for: Recombinant PRV Expressing GP3 and GP5 of PRRSV Provides Effective Protection Against Coinfection With PRV and PRRSV
Source: Transbound Emerg Dis. 2025 May 8;2025:4612568. doi: 10.1155/tbed/4612568 (PMC12081148; doi:10.1155/tbed/4612568)
Supplement: Supporting Information 6 — Table S1: Sequences of sgRNAs and primers for recombinant transfer plasmids construction. [file 4612568.f6.doc]

| Primers | Sequence(5’-3’) |
| --- | --- |
| TK LA-F | ATGACCATGATTACGCCAAGCTTAGCACGCTGTGGCCCTC |
| CMV-R | CAGGTTCTGCAGCCACATGGTGGCTCTAGAAGCTCTGCTTATATAGACCT |
| eGFP-F | GAGGAGAATCCTGGCCCAATGGTGAGCAAGGGCGAGGAGCTGTTCA |
| TK RA-R | AACTAGTCAATAATCAATGCCGTCGACAGCGCCATCACCCGGGCCGTGGTGCTCTTGC |
| gI LA-F | ACCATGATTACGCCAAGCTTCGCGATTCCCCCCTCTCTCTCA |
| mCHERRY-F | CGAGGAGAATCCTGGCCCAATGGTGAGCAAGGGCGAGGA |
| gE RA-R | AAAACGACGGCCAGTGAATTCCTAGGAGATGGTACATCGCGGGG |
| GM-CSF-F | ATGTGGCTGCAGAACCTG |
| GM-CSF-R | GTGCACAGCTATTAACCATCTTTTTGACTGGCCCCCAGC |
| GM-CSF-BmaH Ⅰ-R | GTGCACAGCTATTAACCATGGATCCCTTTTTGACTGGCCCCCAGC |
| GMCSF-T2A-R | AGCAGACTTCCTCTGCCCTCTCCACTGCCCTTTTTGACTGGCCCCCAGC |
| T2A-R | AGCAGACTTCCTCTGCCCTCTCCACTGCCCTTTTTGACTGGCCCCCAGC |
| ORF3-F | ATGGTTAATAGCTGTGCACTCC |
| T2A-ORF3-F | GAGGAGAATCCTGGCCCAATGGTTAATAGCTGTGCACTCC |
| ORF3-R | GTCAAGCATTTCCCCAACATCCGCCGTGCGACATTGAGA |
| ORF3-BmaH Ⅰ-R | GTGCACAGCTATTAACCATGGATCCCTTTTTGACTGGCCCCCAGC |
| ORF3-T2A-R | GCAGACTTCCTCTGCCCTCTCCACTGCCCCGCCGTGCGACATTGAGA |
| ORF5-F | ATGTTGGGGAAATGCTTGACC |
| T2A-ORF5-F | GAGGAGAATCCTGGCCCAATGTTGGGGAAATGCTTGACC |
| ORF5-R | AGCAGACTTCCTCTGCCCTCTCCACTGCCGCTAGCTGGACGACCCCATTGTTCCG |
| sgRNA-TK-11-F | CACCGATCCCCGCCCGGAAGCGCGC |
| sgRNA-TK-11-R | AAACGCGCGCTTCCGGGCGGGGATC |
| sgRNA-TK-2-F | CACCGCGGCGCGGGTGGGAGGGGCG |
| sgRNA-TK-2-R | AAACCGCCCCTCCCACCCGCGCCGC |
| sgRNA-gI-10-F | CACCGACGAGCTAAAAGCGCAGCC |
| sgRNA-gI-10-R | AAACGGCTGCGCTTTTAGCTCGTC |
| sgRNA-gE-42-F | CACCGTCCCGGTATTTAAGCGGGGC |
| sgRNA-gE-42-R | AAACGCCCCGCTTAAATACCGGGAC |

Supplementary Table S1

Sequences of sgRNAs and primers for recombinant transfer plasmids construction.
