# Supplementary material for: Recombinant PRV Expressing GP3 and GP5 of PRRSV Provides Effective Protection Against Coinfection With PRV and PRRSV
Source: Transbound Emerg Dis. 2025 May 8;2025:4612568. doi: 10.1155/tbed/4612568 (PMC12081148; doi:10.1155/tbed/4612568)
Supplement: Supporting Information 7 — Table S2: The primes of PCR for detection genetic stability of recombinant PRVs and RT-qPCR for detection of cytokines. [file 4612568.f7.doc]

Supplementary Table S2

The primes of PCR for detection genetic stability ofrecombinant PRVs and RT-qPCR for detection of cytokines.

| Primers | Sequence(5’-3’) |
| --- | --- |
| CMV-443-F | ACCAATGGGCGTGGATAGC |
| GM-CSF-443-R | CTTGTACAGGTTCAGGCGAG |
| ORF3-400-F | GTTGCAGCTTCTTGCACCTT |
| ORF3-400-R | GCACAAATGAGTTGACCCCG |
| ORF5-381-F | GTCAACGCCAACGACAACAG |
| ORF5-381-R | CCAACGGTAGAGTTTGCCCT |
| TK-273-F | CGCACTCTGTTCGACACGGA |
| TK-273-R | GCTGATGTCCCCGACGATGA |
| gI-472-F | TCGCCACCATCGCAGAAGA |
| gE-472-R | CGGGCAGGAACGTCCAGATC |
| IL-2-qF | TGAGCAGGATGGAGAATTACAGG |
| IL-2-qR | GTCCAAGTTCATCTTCTAGGCAC |
| IL-4-qF | CCTGCTCTTCTTTCTCGAATGT |
| IL-4-qR | CTCTCTGTGGTGTTCTTCGTTG |
| IL-6-qF | CCACTTCACAAGTCGGAGGCTTA |
| IL-6-qR | CCAGTTTGGAAGCATCCATCATTTC |
| IL-10-qF | GCTCTTACTGACTGGCATGAG |
| IL-10-qR | CGCAGCTCTAGGAGCATGTG |
| IFN-γ-qF | CTCTTCTTGGATATCTGGAGGAACTGG |
| IFN-γ-qR | AATGACGCTTATGTTGTTGCTGATGG |
| β-actin-qF | TTGGGAGGGTGAGGGACT |
| β-actin-qR | GAACGGTGAAGGCGACAG |
